# Study on the determinants of health professionals’ performance on diabetes management care in China

**DOI:** 10.1186/s12875-023-02136-z

**Published:** 2023-09-02

**Authors:** Shanshan Jing, Yahang Yu, Beibei Yuan

**Affiliations:** 1 College of Health Sciences, Shandong University of Traditional Chinese Medicine, 4655 Da Xue Road, University Science Park, Changqing District, Jinan, 250355 Shandong China; 2 China Center for Health Development Studies, Peking University, Xue Yuan Road 38, Haidian District, Box 505, Beijing, 100191 China

**Keywords:** Determinants, Health professional, Performance, Diabetes management care

## Abstract

**Background:**

As the direct providers of diabetes management care in primary health care facilities (PHFs) in China, health professionals’ performance on management care of diabetes determines the quality of services and patients’ outcomes. This study aims to analyze the key determinants of health professionals’ performance on diabetes management care in PHFs in China.

**Methods:**

We conducted a cross-sectional study in 72 PHFs in 6 cities that piloted the contracted family doctor service (CFDS). Self-developed questionnaire was used to measure three kinds of factors (capacity, motivation and opportunity) potentially influencing the performance of health professionals. The performance of diabetes management care in the study was measured as whether health professionals delivered 7 service items required by the National Basic Public Health Service Guideline with a total of 7 points and was divided into three grades of good, medium and bad. The questionnaire is self-administered by all the health professionals involved in the study with the number of 434. The Chi-square tests were used to compare differences of performance on diabetes management care among health professionals with different characteristics. The ordinal logistic regression was used to analyze the determinants on the performance of diabetes management care.

**Results:**

Health professionals who got higher score on diabetes knowledge test had odds of better performance on diabetes management care (OR = 1.529, P < 0.001). health professionals with higher degree of self-reported satisfaction on training (OR = 1.224, P < 0.05) and perception of decreasing workload (OR = 3.336, P < 0.01) had odds of better performance on diabetes management care. While health professionals with negative feeling on information system support had odds of worse performance on diabetes management care (OR = 0.664, P < 0.01).

**Conclusions:**

Attention should be paid to the training of health professionals’ knowledge on diabetes management capacity. Furthermore, measures to improve training for health professionals could satisfying their needs for self-growth and improve the motivation of health professionals. The information system supporting management care should be improved continuously to improve the health professionals’ working opportunities and decrease the workload.

## Background

Type 2 diabetes mellitus (T2DM) has become as a global public health issue. In China, the prevalence of T2DM is now 11.2%, being higher than the global average level [1]. The cost of T2DM treatment and management care in China is predicted to exceed RMB 360 billion (almost USD 51 billion) annually by 2030 [2]. It is imposing a huge economic burden for both patients and the whole health system in China. However, the public health services in China especially for chronic diseases such as diabetes, still have many problems, such as the standardization and quality of diabetes management services provided by PHFs is not high, and the rate of diabetes patients with blood glucose under control is low [3]. There are some reasons for these problems: at the level of system or organizational arrangements, most PHFs in China set separated departments for the two kinds of services which are public health services and medical services, and they are reimbursed by different financing system [4]; at the organization level, PHFs held strong motivation in providing more medical services other than public health services, which was attributed to the government subsidies on public health services being relatively low; meanwhile, the revenues could be obtained from delivering medical services which were paid from out-of-pocket payment of patients or social insurance reimbursement through fee-for-service method; at the health worker individual level, the awareness and recognition on the importance of preventing diseases in population is not sufficient in doctors and nurses of PHFs. Doctors had stronger willingness in delivering more medical services, although they had to work on public health services under supervision pressure from health administration department [5].

The primary healthcare system was seen as a means of addressing the burden of chronic non-communicable diseases in China government’s Healthy China 2030 plan [6]. In response to such challenges, the Chinese government has committed to a dramatic increase in the capacity building of the primary health care system [7]. The central government introduced a comprehensive healthcare reform plan in 2009 to strengthen the primary healthcare system in both basic medical services and public health service provision. One important measure was the program entitled “Basic Public Health Services” (BPHS) in which government subsidies support PHFs to deliver a defined package of basic health services throughout the country [8]. In urban areas, PHFs are called community health centers and stations; in rural areas they are township health centers and village clinics. This essential health care package focuses on maternal and child health, health management for the elderly and chronic disease patients. The health management care for chronic disease in this program covers health education, improving medication compliance, control risk factors, such as smoking control, alcohol intake and combating obesity [9], which are in line with the recommendations of the World Health Organization for essential packages of interventions for non-communicable diseases by primary care facilities [10].

In a series of measures strengthening primary health system, one trend is to develop “contracted family doctor service” (CFDS) and try to gradually promote the gate-keeping role of primary health care providers. The CFDS package is suggested by national policy guideline and covers the public services package defined by BPHS and basic medical services. Specifically for T2DM, the CFDS cover health education, control risk factors, screening, regular physical check, health document updating and management, prescription of medicines for controlling blood glucose, direction on medication use and compliance, referral to hospitals for uncontrolled blood glucose or complications. All the services are provided by family doctor team led by health professionals who have been registered as General Practitioners (GPs) or have got physician and assistant physician license.

As the main and direct providers of diabetes management in PHFs, health professionals’ performance on management care of diabetes directly affects quality of services and patient outcomes. Based on behavior change wheel (BCW) framework [11], the impacts of individual health professionals on performance of health services delivery are through three major channels: capacity (ability to perform well), motivation (willingness to exert efforts for performance targets), and opportunity (organizational supports for achieving performance targets). Published studies have ever analyzed one group of determinants on performance. For example, diabetes management can be improved through reform of medical education or training of health professionals [12–14]. Performance-related economic incentives on health professionals lead to better diabetes management care delivery process and outcome performance [15]. Using of an electronic diabetes form was associated with improved screening and GPs with high workload recorded fewer micro vascular screening procedures [16, 17]. Some other factors, like female gender, younger age and high attitude score of GPs were also associated with a better diabetes management [18, 19]. Few studies have explored the correlation between the determinants with performance on chronic disease management in a comprehensive perspective at present. The main purpose of this study is in the setting of PHFs of China to analyze the determinants of health professionals’ performance on T2DM management care in a comprehensive way with the guide of BCW theory.

## Methods

### Sampling

A stratified sampling method was applied to select PHFs and health professionals. Firstly, 6 prefectures from 6 provinces throughout the eastern, middle and western regions representing the high-, middle- and low-level economic status and different development stage of primary health system were selected. Second, we randomly selected 2 districts or counties in each sampled prefecture. Third, we randomly selected 6 community health care centers in each sampled district and 6 township health care centers in each sampled county based on the institution list given by local agency using computer sampling method. If there were no counties in certain prefecture, 12 community health care centers were randomly selected instead. Finally, 72 PHFs were selected, including 47 community health care centers in the urban areas and 25 township health care centers in the rural areas. All the health professionals in the selected PHFs were recruited in the survey. The inclusion criteria were: (1) The cadre being physician; (2) Participation into teams delivering contracted services in the last year; (3) Voluntary participation with informed consent; (4) On-duty on the investigation day. Each participant completed a self-administered questionnaire independently, with research team being on site to address their questions. The response rate was 100%. Because the chronic disease management care is mainly provided by CFDS team, the correlation analysis in this study were only conducted in the sample of health professionals who reported having participated into CFDS in the last year with the number of 434.

### Measurements

#### The dependent variable

The health professionals’ performance of diabetes management care was set as the dependent variable. The performance of diabetes management care in the study was defined based on the National Basic Public Health Service Guideline (the third edition), which aimed to set up comprehensive service mode of continuous measurements of T2DM, including①screening for T2DM; ②T2DM diagnosis; ③regular treatment; ④diet or exercise guidance; ⑤follow-up visit; ⑥regular examinations for T2DM and its complications; ⑦referral service. The health professionals needed to answer whether these services are being provided (yes/no) in the last year and 1 point assigned for “yes” with a total of 7 points. Based on the extend of health professionals following the guideline, we divided the performance of diabetes management into three grades. If the health professionals provided 5 or more items of services, the performance is rate as good. If 3–5 services were provided, the level of performance is rated as medium. If less than 3 services were provided, the level of performance is rated as bad.

#### The independent variables

1. Demographic and job characteristics.

The health professionals’ gender, degree, training background, qualification and professional title was set as the demographic and job characteristics. 5 questions were set in the questionnaire.

(2) Capacity factors.

In the BCW framework [11], capacity means the individual’s psychological and physical capacity to engage in the activity concerned. It includes having the necessary knowledge and skills. The capacity of professionals’ diabetes management was usually measured by knowledge test with the questions selected from the examination for licensed practitioners [14, 20]. In our study, we designed 7 questions about diabetes management knowledge to measure the diabetes management capacity of the health professionals, including the diagnostic criteria of T2DM; the complications of T2DM; the first choice for treatment of T2DM; the understanding of glycemic index; the drug use; and the measurement of glycosylated hemoglobin. Health professionals get 1 score if they answered right. If the score greater than 5 is considered as high; a score between 3 and 5 is rated as medium, and a score below 3 is rated as low. A higher score indicates better capacity in diabetes management.

(3) Motivation factors.

According to the BCW framework [11], motivation means the individual’s degree of willingness to exert and maintain an effort towards organizational goals. Large number of evidences have suggested the factors related financial and non-financial incentives to maximize health worker’s motivation [21–25]. In China, the systematic review has verified that income, career development through promotion and training, and workload were key factors influencing health workers motivations [26]. Therefore, in our study, the factors related motivation level of health professionals were measured by linkage between income and performance of health services, health professionals’ experience of promotions, the perception on workload, and satisfaction with training received. 4 questions were designed such as “How does performance on CFDS has impacted on your personal income?” with the options of increase, no impact and decrease, in which the assumption was the linkage between increase in income with better performance being able to satisfy needs of health workers on financial (income) or non-financial rewards (promotion and self-growth) and motivate health workers.

(4) Opportunity factors.

According to the BCW framework, either physical or social opportunity to perform well is depended on the supports from environment. The opportunity for the health professionals to perform well is depended on the organizational supports [27, 28]. So, we set the health professionals’ perception on the organizational support from information sharing, device and drug configuration for T2DM as the opportunity factors. 3 questions were designed such as “How does the information sharing about diabetes management and care in your organization?” with the options of very good, relatively good, relatively bad, very bad and have no idea, in which the assumption was the higher perception on these supports meaning the organization providing more opportunities to perform well.

### Statistical analyze

This study tries to analyze how different factors influence the performance of diabetes management care among health professionals. Descriptive statistics in the form of frequencies and percentage were used to describe the characteristics of the health professionals. The Chi-square tests were used to compare differences of performance on diabetes management care among health professionals with different characteristics. The ordinal logistic regression was used to analyze the determinants on the performance of diabetes management care.

All analyses were performed using the statistical package Stata version 14.0. A difference of P < 0.05 was considered to be statistically significant.

## Results

### Basic characteristics of investigated health professionals

This study included 576 health professionals from 72 PHFs in 12 administrative districts in China. As Table 1 shows that 56.42% (n = 325) of the investigated health professionals were female, and more than half have been trained with Western medicine (n = 320), 15.45% and 12.5% were with Traditional Chinese Medicine (n = 89) and preventive medicine (n = 72). 67.53%(n = 389) of the health professionals had bachelor’s degree. 75.35% (n = 434) reported having participated into teams delivering CFDS in the last year.

**Table 1:** General status of the health professionals

Item	n	percentage
Sex		
Male	251	43.58
Female	325	56.42
Age		
<= 35 years	249	43.23
36–40 years	124	21.53
41–45 years	93	16.15
> 45 years	110	19.10
Training specialty		
Western medicine	320	55.56
Traditional Chinese Medicine	89	15.45
Combination of Traditional Chinese Medicine and Western medicine	43	7.47
Preventive medicine	72	12.50
Others	52	9.02
Degree		
Master’s degree or above	52	9.03
Bachelor’s degree	389	67.53
Associate’s degree or below	135	23.44
Qualification		
Practicing physician	393	68.23
Assistant practicing physician	88	15.28
Practicing physician of Traditional Chinese Medicine	92	15.97
Assistant practicing physician of Traditional Chinese Medicine	3	0.52
Professional title		
Vice-senior and above	66	11.46
Intermediate	250	43.40
Beginner	250	43.40
Others	10	1.74
Employment mode		
Formally employed	444	77.08
Others	132	22.92
Years of work experience		
< 10 years	224	38.89
10–20 years	214	37.15
> 20 years	138	23.96
If provided contract services in the last year		
Yes	434	75.35
No	142	24.65

### The correlation between performance on diabetes management care and health professionals’ demographic and job characteristics

Table 2 shows the correlation between individual health professional characteristics with whether they have carried out each item of services defined by BPHS management care. The results showed that females performed better in providing diagnosis (p = 0.004) and referral services (p = 0.029) for diabetes patients compared with male health professionals. It was also found that health professionals with the specialty of preventive medicine had lower percentages in undertaking all the service items (p < 0.005) except follow-up visit. Health professionals with other specialties such as medical technology and others performed worse in providing diagnosis (p < 0.005) and regular treatment (p < 0.005) services. Those with training specialty as Traditional Chinese Medicine performed better in providing diagnosis (p = 0.004), regular treatment(p = 0.01) and referral services (p = 0.002) for diabetes patients compared with practicing physicians.

**Table 2:** The management services for T2DM delivered by health professionals with different personal characteristics

Item	Screening (%)	Diagnosis (%)	Regular treatment (%)	Diet/exercise guidance (%)	Follow-up visit (%)	Complication examination (%)	Referral (%)
Gender							
Male	81.2(134/165)	73.9(122/165)	73.3(121/165)	83.6(138/165)	81.8(135/165)	61.2(101/165)	79.1(129/163)
Female	75.4(181/240)	60.3(144/239)	64.9(155/239)	82.0(196/239)	81.4(193/237)	55.5(132/238)	69.3(165/238)
χ^2^ / P	1.900/0.168	8.132/0.004	3.243/0.072	0.181/0.671	0.010/0.922	1.321/0.250	4.762/0.029
specialty							
Western medicine	85.4(193/226)	74.3(168/226)	78.8(178/226)	85.8(194/226)	83.9(188/224)	65.3(147/225)	80.8(181/224)
Traditional Chinese Medicine	82.4(56/68)	77.9(53/68)	79.4(54/68)	88.2(60/68)	80.9(55/68)	64.7(44/68)	85.3(58/68)
Combination of Traditional Chinese Medicine and western medicine	87.9(29/33)	90.9(30/33)	81.8(27/33)	90.9(30/33)	90.9(30/33)	78.8(26/33)	90.9(30/33)
Preventive medicine	36.2(21/58)	17.2(10/58)	13.8(8/58)	62.1(36/58)	69.0(40/58)	13.8(8/58)	19.0(11/58)
Others^*^	81.0(17/21)	25.0(5/20)	45.0(9/20)	75.0(15/20)	80.0(16/20)	45.0(9/20)	78.9(15/19)
χ^2^ / P	68.599/0.000	96.419/0.000	102.424/0.000	22.669/0.000	8.951/0.062	59.962/0.000	104.678/0.000
Degree							
Master’s degree or above	81.8(36/44)	79.5(35/44)	79.5(35/44)	90.9(40/44)	93.0(40/43)	67.4(29/43)	86.0(37/43)
Bachelor’s degree	77.7(223/287)	64.3(184/286)	68.2(195/286)	82.5(236/286)	81.1(231/285)	58.7(168/286)	73.0(208/285)
Associate’s degree or below	76.0(57/75)	62.7(47/75)	61.3(46/75)	78.7(59/75)	77.3(58/75)	49.3(37/75)	67.6(50/74)
χ^2^ / P	0.554/0.758	4.284/0.117	4.238/0.120	2.934/0.231	4.711/0.095	3.948/0.139	4.835/0.089
Qualification							
Practicing physician	74.8(208/278)	62.1(172/277)	64.3(178/277)	80.1(222/277)	81.1(223/275)	55.4(153/276)	68.5(189/276)
Assistant practicing physician	86.8(46/53)	64.2(34/53)	71.7(38/53)	86.8(46/53)	81.1(43/53)	56.6(30/53)	80.4(41/51)
Practicing/Assistant physician of Traditional Chinese Medicine	82.7(62/75)	80.0(60/75)	80.0(60/75)	89.3(67/75)	84.0(63/75)	68.0(51/75)	86.7(65/75)
χ^2^ / P	4.944/0.145	8.458/0.015	7.090/0.029	4.195/0.123	0.343/0.842	3.864/0.145	11.458/0.003
Professional title							
Vice-senior and above	76.9(40/52)	67.3(35/52)	73.1(38/52)	84.6(44/52)	80.4(41/51)	64.7(33/51)	74.5(38/51)
Intermediate	80.1(146/181)	69.4(125/180)	71.7(129/180)	85.6(154/180)	85.5(153/179)	62.8(113/180)	76.1(137/180)
Beginner	74.5(123/165)	61.2(101/165)	63.6(105/165)	78.8(130/165)	77.0(127/165)	51.5(85/165)	70.6(115/163)
Others^**^	85.7(6/7)	71.4(5/7)	57.1(4/7)	85.7(6/7)	100.0(7/7)	42.9(3/7)	71.4(5/7)
χ^2^ / P	2.146/0.543	2.758/0.430	3.551/0.314	2.965/0.397	5.772/0.123	6.138/0.105	1.401/0.705

* Other specialties including nursing, medical examination, medical technology, stomatology and pharmacy

**The “others” in professional title means the respondents didn’t apply for the professional title

### The correlation between performance on diabetes management care and health professionals’ work capacity

Table 3 shows the correlation between health professionals’ capacity characteristics with each item of services defined by BPHS management care. The results showed that there were statistical differences in all items of the services for T2DM among health professionals with different levels of capacities (p < 0.05). And with further comparisons among any two levels of capacity, it was confirmed that health professionals with high and medium T2DM knowledge test score have undertook more items of service (p < 0.017) than the ones with low T2DM knowledge test score.

**Table 3:** The management services for T2DM delivered by health professionals with different work capacity

Item	Screening (%)	Diagnosis (%)	Regular treatment (%)	Diet/exercise guidance (%)	Follow-up visit (%)	Complication examination (%)	Referral (%)
T2DM knowledge test score							
High	87.9(51/58)	77.6(45/58)	77.6(45/58)	94.8(55/58)	93.1(54/58)	75.9(44/58)	81.0(47/58)
Medium	78.4(239/305)	66.4(202/304)	68.8(209/304)	82.9(252/304)	81.8(247/302)	57.8(175/303)	75.2(227/302)
Low	60.5(26/43)	44.2(19/43)	51.2(22/43)	65.1(28/43)	65.1(28/43)	34.9(15/43)	50.0(21/42)
χ^2^ / P	10.995/0.004	12.540/0.002	8.146/0.017	15.274/0.000	12.921/0.002	17.027/0.000	13.987/0.001

### The correlation between performance on diabetes management care and health professionals’ work motivation

As Table 4 shows, health professionals who got promotion in the past year were more likely to provide regular treatment (p = 0.008) to T2DM patients. The majority of health professionals perceived that the CFDS have increased workload and this perception negatively correlated with the service delivery of diagnosis (p = 0.048), regular treatment (p = 0.010), follow-up visit (p = 0.049), complications examination (p = 0.018) and referral services (p = 0.005) for T2DM patients. At the same time, majority of health professionals perceived that the CFDS had resulted in increase or no change in their income, and the ones who considered an increasing of income were more likely to provide follow-up visit service (p = 0.004). We also investigated the satisfaction on training of health professionals, and the result showed that the positive relationship between training and better performance in regular treatment (p = 0.015), lifestyle guidance (p = 0.045), complications examination (p = 0.000) and referral services (p = 0.013).

**Table 4:** The management services for T2DM delivered by health professionals with different work motivation

Item	Screening (%)	Diagnosis (%)	Regular treatment (%)	Diet/exercise guidance (%)	Follow-up visit (%)	Complication examination (%)	Referral (%)
Promotion							
Yes	77.6(156/201)	68.5(137/200)	74.0(148/200)	86.0(172/200)	81.4(162/199)	60.3(120/199)	76.9(153/199)
No	77.6(149/192)	63.0(121/192)	61.5(118/192)	79.2(152/192)	81.2(155/191)	54.7(105/192)	69.5(132/190)
χ^2^ / P	0.000/0.999	1.307/0.253	7.064/0.008	3.190/0.074	0.004/0.948	1.261/0.262	2.725/0.099
Workload impact of contracted service							
Increase	76.3(267/350)	63.6(222/349)	65.6(229/349)	81.4(284/349)	81.0(281/347)	55.5(193/348)	71.5(248/347)
No impact	88.2(45/51)	80.4(41/51)	86.3(44/51)	92.2(47/51)	88.2(45/51)	74.5(38/51)	90.0(45/50)
Decrease	100.0(1/1)	100.0(1/1)	100.0(1/1)	100.0(1/1)	0.0(0/1)	0.0(0/1)	0.0(0/1)
χ^2^ / P	3.972/0.137	6.092/0.048	9.240/0.010	3.739/0.147	6.043/0.049	7.986/0.018	10.524/0.005
Income impact of contracted service							
Increase	76.0(174/229)	67.2(154/229)	68.6(157/229)	84.7(194/229)	86.8(198/228)	61.4(140/228)	74.6(170/228)
No impact	80.0(128/160)	63.5(101/159)	68.6(109/159)	80.5(128/159)	74.1(117/158)	53.5(85/159)	72.6(114/157)
Decrease	100.0(11/11)	81.8(9/11)	72.7(8/11)	81.8(9/11)	90.9(10/11)	54.5(6/11)	72.7(8/11)
χ^2^ / P	4.037/0.133	1.820/0.402	0.087/0.958	1.189/0.552	10.909/0.004	2.484/0.289	0.189/0.910
Training satisfaction							
Strongly dissatisfied	72.7(16/22)	63.6(14/22)	59.1(13/22)	68.2(15/22)	71.4(15/21)	45.5(10/22)	59.1(13/22)
Partly dissatisfied	62.5(25/40)	47.5(19/40)	47.5(19/40)	70.0(28/40)	70.0(28/40)	30.0(12/40)	56.4(22/39)
Slightly dissatisfied	74.4(67/90)	61.1(55/90)	63.3(57/90)	81.1(73/90)	81.1(73/90)	50.0(45/90)	68.9(62/90)
Slightly satisfied	78.6(92/117)	68.1(79/116)	71.6(83/116)	84.5(98/116)	81.7(94/115)	63.5(73/115)	74.8(86/115)
Partly satisfied	86.0(74/86)	73.3(63/86)	74.4(64/86)	89.5(77/86)	87.2(75/86)	69.8(60/86)	82.4(70/85)
Strongly satisfied	82.0(41/50)	70.0(35/50)	78.0(39/50)	86.0(43/50)	86.0(43/50)	66.0(33/50)	82.0(41/50)
χ^2^ / P	10.272/0.068	9.632/0.086	14.042/0.015	11.342/0.045	7.492/0.187	24.244/0.000	14.477/0.013

### The correlation between performance on diabetes management care and health professionals’ perception on opportunity factors

The result in Table 5 showed that there were statistical differences in all items of the services providing for T2DM among health professionals with different perception on information sharing (p = 0.000) and the device configuration (p = 0.007), and those with better feeling on organizational supports having higher proportion in delivering manage care services.

**Table 5:** The management services for T2DM delivered by health professionals with different perception on opportunity factors

Item	Screening (%)	Diagnosis (%)	Regular treatment (%)	Diet/exercise guidance (%)	Follow-up visit (%)	Complication examination (%)	Referral (%)
Information sharing							
Very good	90.8(79/87)	79.3(69/87)	77.0(67/87)	93.1(81/87)	94.3(82/87)	73.6(64/87)	85.1(74/87)
Relatively good	78.0(198/254)	66.8(169/253)	70.4(178/253)	85.0(215/253)	82.5(207/251)	57.9(146/252)	75.0(189/252)
Relatively bad	80.0(20/25)	64.0(16/25)	64.0(16/25)	80.0(20/25)	80.0(20/25)	48.0(12/25)	64.0(16/25)
Very bad	90.9(10/11)	72.7(8/11)	81.8(9/11)	90.9(10/11)	90.9(10/11)	63.6(7/11)	80.0(8/10)
Have no idea	22.2(4/18)	11.1(2/18)	11.1(2/18)	22.2(4/18)	27.8(5/18)	11.1(2/18)	23.5(4/17)
χ^2^ / P	42.885/0.000	31.665/0.000	32.185/0.000	56.634/0.000	46.621/0.000	26.108/0.000	29.937/0.000
Device configuration							
Adequate	76.9(230/299)	69.5(207/298)	69.5(207/298)	82.9(247/298)	82.5(245/297)	58.6(174/297)	74.7(221/296)
Deficient	80.4(86/107)	55.1(59/107)	64.5(69/107)	82.2(88/107)	79.2(84/106)	56.1(60/107)	69.8(74/106)
χ^2^ / P	0.544/0.461	7.165/0.007	0.899/0.343	0.023/0.880	0.549/0.459	0.204/0.652	0.940/0.332
Drug configuration							
Adequate	75.6(205/271)	65.6(177/270)	66.7(180/270)	82.2(222/270)	83.2(223/268)	61.0(164/269)	71.7(193/269)
Deficient	82.2(111/135)	65.9(89/135)	71.1(96/135)	83.7(113/135)	78.5(106/135)	51.9(70/135)	76.7(102/133)
χ^2^ / P	2.259/0.133	0.006/0.941	0.819/0.365	0.138/0.710	1.318/0.251	3.064/0.080	1.114/0.291

### Ordinal logistic regression on the determinants of health professionals’ performance on diabetes management care

The multivariate analysis results in Table 6 showed that the training specialty as combination of Traditional Chinese Medicine and western medicine or preventive medicine, the T2DM knowledge scores, the satisfaction with training, the perception on workload and perception on information system support were factors being correlated with the performance of health professionals on diabetes management care. (P < 0.05).

**Table 6:** Ordered logistic regression of determinants of health professionals’ performance on diabetes management care

Factor	coef	OR	SE	z	P	95% CI	
Gender-male	0.501	1.650	0.446	1.85	0.064	0.971	2.802
Specialty(Western medicine = 1)							
Traditional Chinese Medicine	0.186	1.205	0.810	0.28	0.782	0.322	4.503
Combination of Traditional Chinese Medicine and western medicine	1.565	4.783	3.715	2.02	0.044	1.044	21.916
Preventive medicine	-2.959	0.052	0.020	-7.56	0.000	0.024	0.111
Others	-1.121	0.326	0.178	-2.06	0.040	0.112	0.949
Qualification (Practicing physician = 1)							
Assistant practicing physician	0.278	1.321	0.587	0.63	0.531	0.553	3.158
Practicing/Assistant physician of Traditional Chinese Medicine	-0.460	0.631	0.440	-0.66	0.509	0.161	2.471
Highest degree	-0.273	0.761	0.223	-0.93	0.351	0.429	1.351
Professional title	-0.003	0.997	0.210	-0.02	0.987	0.660	1.506
T2DM knowledge score	0.425	1.529	0.165	3.94	0.000	1.238	1.889
Training satisfaction	0.202	1.224	0.123	2.02	0.044	1.006	1.490
Professional promotion	-0.050	0.952	0.278	-0.17	0.865	0.537	1.687
Contract workload	1.205	3.336	1.549	2.60	0.009	1.343	8.289
Contract income impact	-0.049	0.952	0.250	-0.18	0.853	0.569	1.595
Information share	-0.410	0.664	0.103	-2.63	0.008	0.489	0.901
Device configuration	-0.247	0.781	0.231	-0.83	0.404	0.437	1.395
Drug configuration	0.502	1.652	0.529	1.57	0.117	0.882	3.094

## Discussion

This study used the investigation data from six cities in China and found that three kinds of determinants of health workers performance were all associated with primary health professionals’ performance on diabetes management care in China: health professionals with higher satisfaction on training provided more items of services required by national guideline, health professionals with higher diabetes knowledge score was associated with better performance on diabetes management care, at the same time health professionals perceiving better support from information sharing system and better availability of adequate equipment performed better in diabetes management services.

This study is designed based on behavior change wheel (BCW) framework, which has the similar implication as several other frameworks regarding the determinants of health workers behaviors [29, 30]. Based on these frameworks, health professional performance is the consequence of three factors: the ability to get the job done (their knowledge, skills and experience to perform the job); motivation to work hard (the extent of efforts on performing better); organizational support or opportunities to do a good job (availability of resources, existence of performance-friendly policies and practices, physical and social environment).

This study found that the higher the health professionals’ diabetes knowledge score is correlated to the more items of services required by national guideline on diabetes management care. Knowledge examination score is one of common methods measuring the capacity of health workers, and have ever been used in dental care, internal medicine care and primary health care [31, 32]. The positive relationship between capacity and work performance has been verified in different countries and in different kinds of services, for example a study of rural general practitioners training on mental health capacity building in Mali indicated that a short mental health training intervention for rural general practitioners improved general practitioners´ knowledge and skills, and resulted in a significant number of new patients being diagnosed and managed [33]; another study on the a capacity-building training program for the early recognition and referral of childhood cancer in North-West Cameroon [34] indicated a significant correlation between the participants’ form of training and their mean score for knowledge about childhood cancer types, signs of childhood cancer and the availability of treatment all together.

Regarding the influence of work motivation on performance of health professionals, the data analysis in this study shows that, in the univariate analysis, those who have experience of being promoted in the past year, and who perceived CFDS bringing income increasing and have higher satisfaction on training undertook more items of diabetes management services. While those who considered CFDS increasing the workload undertook less items of diabetes management services. With further analysis in multivariate analysis, satisfaction on training was correlated to better performance on diabetes management care, while perception on higher workload was correlated to lower performance on delivering diabetes management services. Huge amount number of studies [35–41] have explored the factors being able to motivate primary health workers in China and abroad. One systematic review has synthesized the major motivation factors for primary health workers in China and confirmed the influences of the career development and financial income on motivation. The experiences of being promoted and linkage between income with CFDS directly satisfied the needs of health workers for career development and increased income, and so these factors could motivate better performance on diabetes care. The finding on the negative impact of higher workload on delivering diabetes management services is in consistent with other findings on the high workload as a demotivation factor [16, 42]. The satisfaction on training was found as one important aspect contributing better performance on diabetes management services, which is consistent with previous studies [16, 18, 19, 43, 44]. The mechanism on how being satisfaction with training could contribute to better performance include: training could satisfy health workers’ need for self-growth, at the same training could also help the improvement of work capacity [45].

Organizational support, such as availability of infrastructure and supplies, provides one of the most important opportunities for health workers to perform better. In our study, we found that health professionals perceived high levels of diabetes management information sharing performed better in diabetes management services. In other regions of China, the studies have the similar findings: the effect of management care on patient outcomes was nearly 30% stronger in districts/counties with fully established management information systems compared with districts without information systems [27]; PHFs with the support of information system, including the information sharing of health records and medical records system have a better control of blood glucose in diabetes patients [28]. The studies in other countries also found that whether have the information system support is connected with health workers’ work performance: Carolyn J.Green [46] indicated that a web-based chronic disease management (CDM) was found to be a direct critical success factor that allowed this group of physicians to improve their practice by tracking patient care processes using evidence-based clinical practice guideline-based flow sheets; Griffin and Kinmonth [47] concluded in their Cochrane review that responsibility for diabetes by family physicians will only succeed with adequate support in the office practice such as computerized, prompted recall and review of patients with diabetes.

This study has several limitations. First, the observational nature of our study limited our ability to draw any causal inference from our findings. The health professionals’ performance on diabetes management care maybe influenced by other uncontrollable factors including health system characteristics and other environmental factors. Future studies should focus on more rigorous research, including randomized, controlled trials and observational studies with concurrent control groups, to assess the effectiveness of the relevant policies targeting behaviors of health professionals. Second, in this study, diabetes management services provision was self-reported and may leads to higher performance of diabetes management than what the performance actually is. However, the determinants of health professionals’ performance on diabetes management care and explanation based on BCM theory can still provide in-depth understanding and reliable evidence support for policies targeting improvement of diabetes management care in China.

## Conclusions

Chronic disease management service has gradually become the major tasks of primary health facilities in China. Whether health professionals can provide qualified management services for diabetes patients will directly contribute to health status of diabetes patients and performance of whole primary health system. Based on the findings of this study, attention should be paid to the training of health professionals’ knowledge on diabetes management capacity. Furthermore, measures should be taken to provide satisfactory training for health professionals to improve the motivation of diabetes management. It is also concluded that the information system supporting management care should be improved continuously, which could not only improve the health professionals’ working opportunities for diabetes management but also decrease the damage of high workload on enthusiasm of health professionals on diabetes management services, as poor information system implies lots of manual works in diabetes management care.

## Data Availability

The datasets generated and/or analyzed during the current study are not publicly available due to the nature of a confidentiality agreement with study participants that only members of the study team will have access to the study data. However, for research purposes, de-identifed study data are available from the corresponding author on reasonable request.

## Abbreviations

BCW: Behavior Change Wheel
BPHS: Basic Public Health Services
CFDS: Contracted family doctor service
GP: General practitioner
OR: Odds ratio
PHF: Primary health care facilities
T2DM: Type 2 diabetes mellitus

## References

[1] Li Y, Teng D, Shi X, Qin G, Qin Y, Quan H, Shi B, Sun H, Ba J, Chen B (2020). Prevalence of diabetes recorded in mainland China using 2018 diagnostic criteria from the american Diabetes Association: national cross sectional study. BMJ.

[2] Wang W, McGreevey WP, Fu C, Zhan S, Luan R, Chen W, Xu B (2009). Type 2 diabetes mellitus in China: a preventable economic burden. Am J Manag Care.

[3] Li Y, Teng D, Shi X, Qin G, Qin Y, Quan H (2020). Prevalence of diabetes recorded in mainland China using 2018 diagnostic criteria from the american Diabetes Association: national cross sectional study. BMJ.

[4] Jin Y, Wang H, Wang D, Yuan B (2019). Job satisfaction of the primary healthcare providers with expanded roles in the context of health service integration in rural China: a cross-sectional mixed methods study. Hum Resour Health.

[5] Yu M, Zhao X, Li H, Yu Y, Yuan B, Meng Q (2021). Perception and evaluation of integrated medical and preventive services among primary care doctors and nurses in China. Chin Gen Pract.

[6] Li X, Krumholz HM, Yip W, Cheng KK, Maeseneer JD, Meng Q, Mossialos E, Li C, Lu J, Su M (2020). Quality of primary health care in China: challenges and recommendations. Lancet.

[7] Li X, Lu J, Hu S, Cheng KK, De Maeseneer J, Meng Q, Mossialos E, Xu DR, Yip W, Zhang H (2017). The primary health-care system in China. Lancet.

[8] Yip WC-M, Hsiao WC, Chen W, Hu S, Ma J, Maynard A (2012). Early appraisal of China’s huge and complex health-care reforms. Lancet.

[9] Toward a healthy and harmonious life in China: stemming the rising tide of non-communicable diseases. Washington DC: World Bank; 2011.

[10] Package of essential non-communicable (PEN) disease interventions for primary health care in low-resource settings. Geneva: World Health Organization; 2010.

[11] Michie S, Stralen MMv, West R (2011). The behaviour change wheel: a new method for characterising and designing behaviour change interventions. Implement Sci.

[12] Bhalla S, Unnikrishnan R, Srivastava R, Tandon N, Mohan V, Prabhakaran D (2016). Innovation in capacity building of primary-care physicians in diabetes management in India: a new slant in medical education. Lancet Diabetes Endocrinol.

[13] Murugesan N, Shobana R, Snehalatha C, Kapur A, Ramachandran A (2009). Immediate impact of a diabetes training programme for primary care physicians-an endeavor for national capacity building for diabetes management in India. Diabetes Res Clin Pract.

[14] Li Y, Yan Y, Dai H, Cheng Y, Huang Q, Shao J, Zhou J, Wang H, Liu P, Shen A, Mi Y, Du Z (2021). Mastery of type 2 diabetes prevention and treatment knowledge by general practitioners in Shanghai: a cross-sectional study. BMC Fam Pract.

[15] Jin Y, Tian W, Yu Y, Pan W, Yuan B (2022). Incentives promoting contracted Family Doctor Service Policy to Improve Continuity and Coordination in Diabetes Patient Management Care in China. Front Public Health.

[16] Bakke Ã, Tran AT, Dalen I, Cooper JG, Løvaas KF, Jenum AK, Berg TJ, Madsen TV, Nøkleby K, Gjelsvik B (2019). Population, general practitioner and practice characteristics are associated with screening procedures for microvascular complications in type 2 diabetes care in Norway. Diabet Med.

[17] Varroud-Vial M (2011). Improving diabetes management with electronic medical records. Diabetes Metab.

[18] Ferroni E, Casotto V, Pigato M, Scroccaro G, Corti MC, Fedeli U, Saugo M (2016). Patient and general practitioner characteristics influencing the management of non-insulin-treated diabetes mellitus: a cross-sectional study in Italy. Diabetes Res Clin Pract.

[19] Tran AT, Bakke Ã, Berg TJ, Gjelsvik B, Mdala I, Nøkleby K, Rai AS, Cooper JG, Claudi T, Løvaas K (2018). Are general practitioners characteristics associated with the quality of type 2 diabetes care in general practice? Results from the norwegian ROSA4 study from 2014. Scand J Prim Health Care.

[20] Wei MH, Chen XZ, Zhan XX, Zhang ZX, Yu SJ, Yan WR (2019). The effect of a web-based training for improving primary health care providers’ knowledge about diabetes mellitus management in rural China: a pre-post intervention study. PLoS ONE.

[21] Agyepong IA, Anafi P, Asiamah E, Ansah EK, Ashon DA, Narh-Dometey C (2004). Health worker (internal customer) satisfaction and motivation in the public sector in Ghana. Int J Health Plann Manage.

[22] Franco LM, Bennett S, Kanfer R, Stubblebine P (2004). Determinants and consequences of health worker motivation in hospitals in Jordan and Georgia. Soc Sci Med.

[23] Hotchkiss DR, Banteyerga H, Tharaney M (2015). Job satisfaction and motivation among public sector health workers: evidence from Ethiopia. Hum Resour Health.

[24] Mathauer I, Imhoff I (2006). Health worker motivation in Africa: the role of non-financial incentives and human resource management tools. Hum Resour Health.

[25] Willis-Shattuck M, Bidwell P, Thomas S, Wyness L, Blaauw D, Ditlopo P (2008). Motivation and retention of health workers in developing countries: a systematic review. BMC Health Serv Res.

[26] Li H, Yuan B, Wang D, Meng Q (2019). Motivating factors on performance of primary care workers in China: a systematic review and meta- analysis. BMJ Open.

[27] Qin J, Zhang Y, Fridman M, Sweeny K, Zhang L, Lin C, Mao L (2021). The role of the Basic Public Health Service program in the control of hypertension in China: results from a cross-sectional health service interview survey. PLoS ONE.

[28] Zhao X, Yu M, Yu Y, Li H, Yuan B, Meng Q (2021). Effect of primary operating environment on glycemic control of diabetic patients with type 2 diabetes in an integrated health service system reform. Chin J Health Policy.

[29] Franco LM, Bennett S, Kanfer R (2002). Health sector reform and public sector health worker motivation: a conceptual framework. Soc Sci Med.

[30] Kanungo R, Mendonca M (1994). Work motivation: models for developing countries.

[31] AlFayyad IN, Al-Tannir MA, AlEssa WA, Heena HM, Abu-Shaheen AK (2019). Physicians and nurses’ knowledge and attitudes towards advance directives for cancer patients in Saudi Arabia. PLoS ONE.

[32] Tahani B, Manesh SS (2021). Knowledge, attitude and practice of dentists toward providing care to the geriatric patients. BMC Geriatr.

[33] Poudiougou O, Bruand P-E, Mounkoro PP, Gaglione J-M, Nimaga K, Sy M, Vincent C, Calas F, Fall-Ndao A, Petiteau L (2021). Mental health capacity building in Mali by training rural general practitioners and raising community awareness. Pan Afr Med J.

[34] Afungchwi GM, Hesseling PB, Kouya F, Enow SA, Kruger M (2020). The outcome and cost of a capacity-building training programme on the early recognition and referral of childhood cancer for healthcare workers in North-West Cameroon. Nurs Open.

[35] Ebenso B, Mbachu C, Etiaba E, Huss R, Manzano A, Onwujekwe O, Uzochukwu B, Ezumah N, Ensor T, Hicks JP, Mirzoev T (2020). Which mechanisms explain motivation the of primary health workers? Insights from the realist evaluation of a maternal and child health programme in Nigeria. BMJ Glob Health.

[36] Gupta J, Patwa MC, Khuu A, Creanga AA (2021). Approaches to motivate physicians and nurses in low- and middle-income countries: a systematic literature review. Hum Resour Health.

[37] Jiang H, Jia H, Zhang J, Li Y, Song F, Yu X (2021). Nurses’ occupational stress and presenteeism: the mediating role of Public Service Motivation and the moderating role of Health. Int J Environ Res Public Health.

[38] Li L, Hu H, Zhou H, He C, Fan L, Liu X, Zhang Z, Li H, Sun T (2014). Work stress, work motivation and their effects on job satisfaction in community health workers: a cross-sectional survey in China. BMJ Open.

[39] Millar R, Chen Y, Wang M, Fang L, Liu J, Xuan Z, Li G (2017). It’s all about the money? A qualitative study of healthcare worker motivation in urban China. Int J Equity Health.

[40] Zhang P (2014). Reform of public hospitals and the main role of medical staffs. Cell Biochem Biophys.

[41] Weldegebriel Z, Ejigu Y, Weldegebreal F, Woldie M (2016). Motivation of health workers and associated factors in public hospitals of West Amhara, Northwest Ethiopia. Patient Prefer Adherence.

[42] Woolley A, Li L, Solomon J, Li J, Huang K, Chahal P, Chahal P, Tu G, Chahal P, Chattopadhyay K (2020). What are the development priorities for management of type 2 diabetes by general practitioners in Ningbo, China: a qualitative study of patients’ and practitioners’ perspectives. BMJ Open.

[43] Puder JJ, Keller U (2003). Quality of diabetes care: problem of patient or doctor adherence?. Swiss Med Wkly.

[44] Yao M, Zhang D-Y, Fan J-T, Lin K, Haroon S, Jackson D, Li H, Chen W, Lehman R, Cheng KK (2021). The experiences of chinese general practitioners in communicating with people with type 2 diabetes-a focus group study. BMC Fam Pract.

[45] Zhao X, Wang H, Li J, Yuan B (2020). Training primary healthcare workers in China’s township hospitals: a mixed methods study. BMC Fam Pract.

[46] Green CJ, Fortin P, Maclure M, Macgregor A, Robinson S (2006). Information system support as a critical success factor for chronic disease management: necessary but not sufficient. Int J Med Inform.

[47] Griffin S, Kinmonth AL (2000). Diabetes care: the effectiveness of systems for routine surveillance for people with diabetes. Cochrane Database Syst Rev.

